# Planting Trees

**Published:** 1858-02

**Authors:** 


### PLANTING TREES.

THE following table shows in feet what number of trees can be planted in one acre of ground, that is, in forty-three thousand five hundred and sixty square feet (43,560). It is done by multiplying the distances by one another, and dividing the product into 43,560. Thus, if one plant is distant from another one foot each way, an acre will contain 43,560 plants; if forty feet apart, the proper distance for apple-trees, then an acre will contain twenty-seven apple-trees. A good apple-tree will yield fifty bushels, making thirteen hundred and fifty bushels of apples to a single acre. With such a result, who can tell why it is that such a delicious, useful, and healthful fruit, is cultivated to so limited an extent, that a bushel can seldom be purchased in New York for less than one dollar!

| Dist. apart. | No. of Plants. | Dist. apart. | No. of Plants. |
|---|---|---|---|
| 1 by 1 | 43,560 | 7 by 7 | 888 |
| 1½ " 1½ | 19,360 | 8 " 8 | 680 |
| 2 " 1 | 21,780 | 9 " 9 | 537 |
| 2 " 2 | 10,890 | 10 " 10 | 435 |
| 2½ " 2½ | 6,969 | 11 " 11 | 360 |
| 3 " 1 | 14,520 | 12 " 12 | 302 |
| 3 " 2 | 7,260 | 13 " 13 | 257 |
| 3 " 3 | 4,840 | 14 " 14 | 222 |
| 3½ " 3½ | 3,555 | 15 " 15 | 193 |
| 4 " 1 | 10,890 | 16 " 16 | 170 |
| 4 " 2 | 5,445 | 17 " 17 | 150 |
| 4 " 3 | 3,630 | 18 " 18 | 134 |
| 4 " 4 | 2,722 | 19 " 19 | 120 |
| 4½ " 4½ | 2,151 | 20 " 20 | 108 |
| 5 " 1 | 8,712 | 24 " 24 | 75 |
| 5 " 2 | 4,356 | 25 " 25 | 69 |
| 5 " 3 | 2,904 | 27 " 27 | 59 |
| 5 " 4 | 2,178 | 30 " 30 | 48 |
| 5 " 5 | 1,742 | 40 " 40 | 27 |
| 5½ " 5½ | 1,417 | 50 " 50 | 17 |
| 6 " 6 | 1,210 | 60 " 60 | 12 |
| 6½ " 6½ | 1,031 | 66 " 66 | 10 |

